# Association between Dipeptidyl Peptidase-4 Inhibitors and Allergic Rhinitis in Asian Patients with Diabetes

**DOI:** 10.3390/ijerph16081323

**Published:** 2019-04-12

**Authors:** Hsin-Hung Chen, Shang-Yi Li, Weishan Chen, Chia-Hung Kao

**Affiliations:** 1 Institute of Medicine and Public Health, Chung Shan Medical University, Taichung 402, Taiwan; 605319@cch.org.tw; 2 College of Nursing and Health Sciences, Dayeh University, Changhua 515, Taiwan; 3 Division of Metabolism Endocrinology, Changhua Christian Hospital, Changhua 500, Taiwan; 4 Division of Metabolism Endocrinology, Nantou Christian Hospital, Nantou 540, Taiwan; 5 Department of Otorhinolaryngology, Head and Neck Surgery, Changhua Christian Hospital, Changhua 500, Taiwan; 150614@cch.org.tw; 6 Management Office for Health Data, China Medical University Hospital, Taichung 404, Taiwan; sandy8121985@gmail.com; 7 College of Medicine, China Medical University, Taichung 404, Taiwan; 8 Graduate Institute of Biomedical Sciences, College of Medicine, China Medical University, Taichung 404, Taiwan; 9 Department of Nuclear Medicine and PET Center, China Medical University Hospital, Taichung 404, Taiwan; 10 Department of Bioinformatics and Medical Engineering, Asia University, Taichung 413, Taiwan

**Keywords:** dipeptidyl peptidase-4 (DPP-4) inhibitor, allergic rhinitis, diabetes

## Abstract

In this retrospective study, we attempted to evaluate the association between dipeptidyl peptidase-4 (DPP-4) inhibitors and allergic rhinitis in patients with diabetes. We analyzed the Longitudinal Health Insurance Database 2000 subdatabase. Our study population included patients with type 2 diabetes (ICD-9-CM 250) between 2009 and 2012, and the study groups were DPP-4 inhibitor users and nonusers. Propensity scores were estimated in a multivariable logistic regression model for the analysis of allergic rhinitis (ICD-9-CM 477.9). Each group consisted of 6204 patients. DPP-4 inhibitor users had a reduced risk of allergic rhinitis (aHR = 0.74, 95% confidence interval (CI) = 0.61–0.90) in all stratifications. Among women, DPP-4 inhibitor users had a lower risk of allergic rhinitis (aHR = 0.67, 95% CI = 0.50–0.90). Among patients aged older than 40 years, DPP-4 inhibitor users had a decreased risk of allergic rhinitis (those aged 40–59: aHR = 0.75, 95% CI = 0.56–0.99; those aged ≧60: aHR = 0.73, 95% CI = 0.54–0.97). Among patients with comorbidities, the risk of allergic rhinitis for DPP-4 inhibitor users was 0.73 (95% CI = 0.60–0.90). High-dose (cumulative defined daily dose ≧648mg) DPP-4 inhibitor users had a decreased risk of allergic rhinitis (aHR = 0.23, 95% CI = 0.15–0.35). Our study revealed that Asian patients with diabetes who used DPP-4 inhibitors had decreased risk of allergic rhinitis, especially for DPP-4 inhibitor treatment in patients who were women, were older than 40 years, had higher diabetes severity scores, were taking higher doses of DPP-4 inhibitors, and had diabetes with comorbidities.

## 1. Introduction

A study showed that compared with patients without diabetes who had acute myocardial infarction (AMI), patients with diabetes have the same risk of suffering an AMI attack [1]. Other studies have shown that patients with diabetes have a risk between 2-and 4-fold higher of developing cardiovascular disease, which may result in a more than 3-fold greater risk of mortality [2,3,4]. In 2011, diabetes mellitus (DM) was one of the critical non-communicable diseases emphasized by the World Health Organization (WHO), which has released numerous public health policies for DM control. DM is also a crucial public health problem in Taiwan and thus remains among the 10 leading causes of death. Many guidelines exist for medications used to treat DM. Dipeptidyl peptidase-4 (DPP-4) inhibitors are a popular oral antidiabetic agent (OAD) [5]. Relevant studies have reported higher rates for adverse events such as nasopharyngitis, arthralgia, and headache related to DPP-4 inhibitor adverse events; however, regarding comparisons with other OADs, no findings have been statistically significant [6,7]. The DPP family has been observed in multiple organs and tissues, including some involved in the immune system, which could potentially lead to immune-modulating effects after taking DPP-4 inhibitors [8,9]. Prior to our research on DPP-4 inhibitors and allergic rhinitis (AR), we found a previous study talked about the effect of DPP-4 inhibitors on asthma control [10]. In the article, there were many mechanisms showed to explain the immunological pathways of DPP-4 inhibitors such as CD26, and it was also mentioned that there were many DPP-4 transcripts in the nasal epithelia of children who suffered from dust mite AR. AR is another prevalent disease worldwide, and many studies have mentioned that the major mechanism of AR is the stimulation of specific immunoglobulin E (IgE) [11,12]. Based on the immune mechanisms between AR and DPP-4 inhibitor, we hypothesized that some adverse events of nasopharyngitis caused by DPP-4 inhibitors may be related to AR attacks.

## 2. Methods

### 2.1. Data Source

Taiwan’s National Health Insurance program was established in 1995 and covers more than 99% of Taiwan’s population. In this study, we analyzed the Longitudinal Health Insurance Database 2000 subdatabase, from which we randomly selected 1 million patients listed in the National Health Insurance Research Database. Patient identification numbers were re-encoded when using the database to obtain patients’ demographic information, inpatient and outpatient records, medications, and treatments, while protecting their privacy. This study was approved by the Institutional Review Board of China Medical University as well as the Hospital Research Ethics Committee (IRB permit number: CMUH104-REC2-115-CR3).

### 2.2. Sampled Participants

Our study population included patients newly diagnosed with type II DM (ICD-9-CM 250) between 2009 and 2012; the study group comprised patients with DM who had received DPP-4 inhibitors, and the index date was the date of initial DPP-4 inhibitor therapy. We excluded patients who received DPP-4 inhibitors before being diagnosed with DM, had a history of AR (ICD-9-CM 477.9), or withdrew from the insurance program before the index date. The controls were randomly selected patients with DM who had never received DPP-4 inhibitor and were randomly assigned an index date between the date of DM diagnosis and December 31, 2012. The exclusion criteria for controls were the same as those for DPP-4 inhibitor users. Subsequently, the control group was one-fold size matched with the DPP-4 inhibitor group using propensity score matching. Furthermore, propensity scores were estimated in a multivariable logistic regression model with the following variables: DM diagnosis year, index year, gender, diabetes complications severity index (DCSI) score, and the comorbidities of coronary artery disease (CAD; ICD-9-CM 410–414), stroke (ICD-9-CM 430–438), hypertension (ICD-9-CM 401–405), hyperlipidemia (ICD-9-CM 272), and chronic kidney disease (CKD; ICD-9-CM 580–589). To evaluate the severity of DM, the DCSI score was based on diabetic complications. We also considered oral antidiabetic agents including thiazolidinedione, sulfonylureas, biguanides, and other antidiabetic drugs.

### 2.3. Events and Comorbidities

The event discussed in the present study was AR (ICD-9-CM 477.9). The end of the study was marked by the occurrence of AR, withdrawal from the insurance program, or the end of 2013. We considered CAD, stroke, hypertension, hyperlipidemia, and CKD to be risk factors for AR.

### 2.4. Statistical Analysis

Table 1 presents the demographic characteristics, DCSI scores, and comorbidities of DPP-4 inhibitor users and nonusers. We described the continuous variable of age using a mean and standard deviation and evaluated the difference between the two groups using the Wilcoxon rank-sum test. We presented the categorical variables as numbers and percentages and tested the differences between groups using chi-squared tests. Furthermore, the hazard ratios (HRs) and 95% confidence intervals (CIs) of the two groups were estimated using univariate and multivariate Cox proportional-hazards regression models. The variables in the multivariate model were gender, age, DCSI score, and comorbidities. Table 2 presents the HR of AR with the dosage of DPP-4 inhibitors. Moreover, we describe the DPP-4 inhibitor dosage using the cumulative defined daily dose (cDDD). According to the WHO, the cDDD is a unit of measurement for drugs and could help us to compute the doses of multiple drug types. Next, we divided DPP-4 inhibitor users into three groups by the first, second, and third tertiles of the cDDD. We used multiplicative analysis to evaluate the interaction effect of DPP-4 inhibitors and other oral antidiabetic agents on AR risk. Kaplan–Meier survival curves were used to describe the cumulative incidence of AR for the two groups, and the differences between the two groups were tested using a log rank test. The data analysis in this study was performed using SAS statistical software (Version 9.4 for Windows; SAS Institute, Inc., Cary, NC, USA); *p* <0.05 was considered statistically significant.

## 3. Results

Each group had 6204 patients with similar demographics; approximately 42% were women, and the mean age was 58.6 years (Table 1). Compared to the nonusers, the DPP-4 inhibitor users had a higher prevalence of other oral antidiabetic agents, including thiazolidinedione, sulfonylureas, biguanides, and other antidiabetic drugs (all *p*-values <0.001). The mean (range) follow-up period in DPP-4 inhibitor users and nonusers were 2.45 (0.01–4.86) and 2.37 (0.003–4.85) years, respectively.

**Figure 1:**
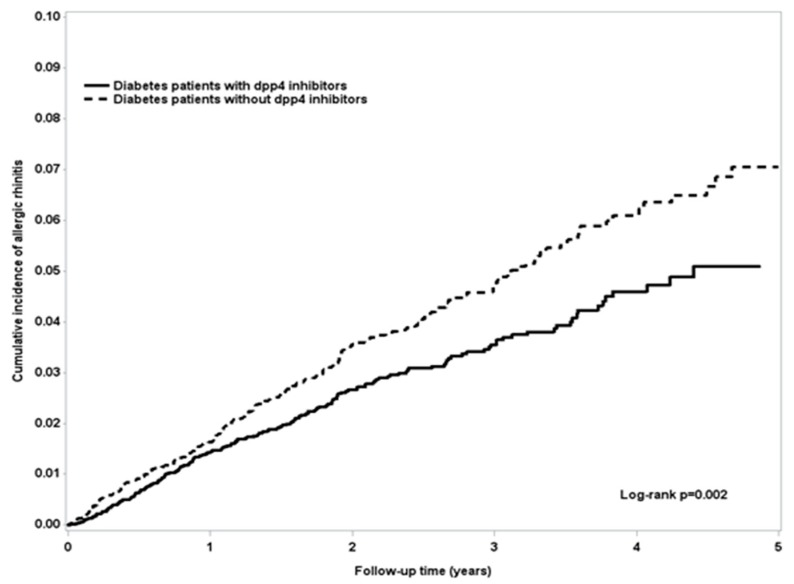
The incidence rates of allergic rhinitis (AR) for DPP-4 inhibitor users and nonusers were 1.24 and 1.66 per 100 person-years, respectively. DPP-4 inhibitor users exhibited a reduced risk of AR (aHR = 0.74, 95% confidence interval (CI) = 0.61–0.90), and DPP-4 users had a lower incidence of AR.

The incidence rates of AR for DPP-4 inhibitor users and nonusers were 1.24 and 1.66 per 100 person-years, respectively. DPP-4 inhibitor users exhibited a reduced risk of AR (aHR = 0.81, 95% CI = 0.73–0.90) (Table 2), and DPP-4 inhibitor users had a lower incidence of AR, as evidenced in Figure 1. Next, we classified patients by gender, age, DCSI score, and comorbidities, and patients with any comorbidity were classified as the comorbidity group. Across all stratifications, we found that DPP-4 inhibitor users had a lower incidence rate of AR than did nonusers (among women: aHR = 0.74, 95% CI = 0.63–0.88; those aged 40–59 years: aHR = 0.83, 95% CI = 0.71–0.96; those aged ≧60 years: aHR = 0.78, 95% CI = 0.66–0.91; those with a DCSI score ≧4: aHR = 0.70, 95% CI = 0.57–0.85; and those with a comorbidity: aHR = 0.78, 95% CI = 0.70–0.87).

Subsequently, we discussed the dose of DPP-4 inhibitors, the values for which are presented in Table 3. Compared with nonusers, low-dose (cDDD ≦279 mg) DPP-4 inhibitor users had a 1.87-fold higher risk of AR (95% CI = 1.65–2.11), medium-dose (280 ≦ cDDD ≦ 647 mg) DPP-4 inhibitor users had a lower risk of AR (aHR = 0.75, 95% CI = 0.64–0.88), and high-dose (cDDD ≧ 648 mg) DPP-4 inhibitor users had a lower risk of AR (aHR = 0.25, 95% CI = 0.20–0.31).

We observed statistically significant lower risk for patients with both DPP-4 inhibitors and Thiazolidinedione (aHR = 0.69, 95% CI = 0.56–0.85), both with DPP-4 inhibitors and sulfonylureas (aHR = 0.76, 95% CI = 0.59–0.99) or both with DPP-4 inhibitors and other antidiabetic drugs (aHR = 0.63, 95% CI = 0.52–0.76) than those without DPP-4 inhibitors, thiazolidinedione, sulfonylureas, and other antidiabetic drugs (Table 4).

## 4. Discussion

### 4.1. DM and AR

In terms of quality of life, the effect of AR on patients is obvious. In the United States, the cost of AR treatment is estimated to be more than US$6 billion per year [13,14]. One of the key methods for managing AR is to reduce exposure to sensitizing allergens [15,16]. Avoiding possible allergens can decrease specific immunoglobulin E (IgE)-mediated reactions, which are driven by type 2 helper T (Th2) cells. The chain reaction of AR could cause eosinophils and basophils aggregation at the same time as mucosal inflammation [11,12]. IgE induced the reaction of T-cells, B-cells, and mast cells, which resulted in more cytokine (such as interleukin (IL)-4 and IL-18) stimulation in patients with AR [17]. DM with insulin resistance (IR) altered lipid homeostasis and cytokines. IR might result in higher levels of inflammatory markers, such as C-reactive protein and IL-6, because of increased systemic inflammation [18]. A study showed that DM was associated with allergic states [19]. Through modified innate immunity, patients with DM had a higher incidence of impaired endothelial function and higher rate of coagulopathy dysfunction caused by a prothrombotic condition [20,21]. Compared with other Asian studies [22,23], type 2 DM was not a substantial risk factor for AR. For example, DM with comorbidities was not a risk factor for AR in a Korean study, and rheumatic arthritis was a risk factor for AR in a Taiwanese study. In the present study, patients with diabetes and DPP-4 inhibitors had a lower incidence rate of AR. Patients with diabetes with comorbidities such as cardiovascular disease, stroke, or hypertension had lower AR incidence rates in the DPP-4 inhibitor group. Lower AR incidence rates were noted in our female patients with DM who used DPP-4 inhibitors. The severity of DM is a factor for AR attacks, but a lower AR incidence rate was noted in the DPP-4 inhibitor group. Overall, our study showed that DPP-4 inhibitors might play a role in decreased AR incidence rates.

### 4.2. DPP-4 Inhibitor Immunity and AR

In our analysis, higher cDDDs of DPP-4 inhibitors could decrease AR. A possible explanation is that a higher dose of DPP-4 inhibitors could alter the immune response of patients with DM. Studies have mentioned the relationship between DPP-4 inhibitors and infection [7,24], asthma [25], and lung injury [26]. Cluster of differentiation (CD) 26 might play a crucial role between DPP-4 inhibitors and immune regulation [27,28]. Higher levels of CD26 were equal to strongly activated T-cells and might cause potential immune-modulating effects in patients with DM [8]. However, DPP-4 inhibitors belong to the DPP-4 family, and some studies have shown that DPP-4 inhibitors could affect DPP-8 and DPP-9 reactions and thereby induce more immune-modulating effects [9]. In one in vitro study, DPP-4 inhibitor could modify T-cell function and decrease the production of inflammatory cytokines [29]. Activated T-cells were also noted in the pathway of the AR mechanism induced by IgE cells [17]. Higher doses of DPP-4 inhibitor (>280 cDDD) could alter the T-cell pathway to improve the IgE response in AR. To confirm this, more detailed basic animal or cell studies could be designed and conducted in the future.

## 5. Limitations

This study had some limitations. First, in our database, we could not determine all other potential confounding factors, including patients’ nutritional states, smoking or alcohol consumption, and environmental factors such as particular matter(PM)2.5. Second, our study group only included type 2 DM because DPP-4 inhibitors are prescribed to patients with this disease in Taiwan. Finally, AR diagnoses were only collected according to ICD9 codes and without any confirmation from other specialists.

## 6. Conclusions

Few studies have discussed the association between DPP-4 inhibitors and AR. Our study revealed that Taiwanese patients with DM who used DPP-4 inhibitors exhibited decreased incidence rates of AR, especially in the cases of DPP-4 inhibitor treatment in women, patients older than 40 years, those with higher DCSI scores, and those with DM and comorbidities.

## Figures and Tables

**Table 1:** Baseline characteristics of diabetes patients with dipeptidyl peptidase-4 (DPP-4) inhibitors or not.

Variable	Diabetic Patients with DPP-4 inhibitors				*p*-Value *
	Non-Users		Users		
	(*n* = 6204)		(*n* = 6204)		
	*n*	%	*n*	%	
Gender					0.51
Female	2627	42.3	2663	42.9	
Male	3577	57.7	3541	57.1	
Age, years					0.0002
20–39	531	8.56	419	6.75	
40–59	2959	47.7	3103	50.0	
≧60	2714	43.7	2682	43.2	
Mean (SD)	58.4 (13.5)		58.6 (12.4)		0.60 ^a^
DCSI score					0.39
0–1	2432	39.2	2373	38.2	
2–3	2036	32.8	2031	32.7	
≧4	1736	28.0	1800	29.0	
Comorbidity					
CAD	1934	31.2	1962	31.6	0.59
Stroke	576	9.28	607	9.78	0.34
Hypertension	4252	68.5	4314	69.5	0.23
Hyperlipidemia	4279	69.0	4370	70.4	0.08
CKD	917	14.8	918	14.8	0.98
Medication					
Thiazolidinedione	314	5.06	971	15.7	<0.001
Sulfonylureas	638	10.3	974	15.7	<0.001
Biguanides	902	14.5	1146	18.5	<0.001
Other antidiabetic drugs	491	7.91	1425	23.0	<0.001

*: chi-squared test; ^a^: Wilcoxon’s rank-sum test. CAD: coronary artery disease; CKD: chronic kidney disease; DSCI score: diabetes complications severity index.

**Table 2:** Incidence rate and hazard ratio of allergic rhinitis in diabetes patients of DPP-4 inhibitor users compared to non-users.

Variable	Diabetic Patients with DPP-4 inhibitors								Compared to Non-User	
	Non-Users			Users			Crude		Adjusted	
	Event	PY	IR	Event	PY	IR	HR (95% CI)	*p*-Value	HR (95% CI)	*p*-Value
Overall	245	14,725	1.66	188	15,219	1.24	0.75 (0.62, 0.91)	0.003	0.81 (0.73, 0.90)	0.001
Gender										
Female	111	6308	1.76	77	6659	1.16	0.67 (0.50, 0.90)	0.008	0.74 (0.63, 0.88)	0.004
Male	134	8417	1.59	111	8560	1.30	0.81 (0.63, 1.05)	0.11	0.87 (0.75, 1.00)	0.05
Age										
20–39	29	1280	2.27	21	1061	1.98	0.86 (0.49, 1.51)	0.60	0.84 (0.56, 1.26)	0.39
40–59	111	7283	1.52	88	7898	1.11	0.75 (0.56, 0.99)	0.045	0.83 (0.71, 0.96)	0.01
≧60	105	6163	1.70	79	6260	1.26	0.74 (0.55, 0.996)	0.047	0.78 (0.66, 0.91)	0.03
DCSI score										
0–1	90	5866	1.53	73	5799	1.26	0.83 (0.61, 1.14)	0.24	1.02 (0.86, 1.21)	0.85
2–3	74	4931	1.50	56	5110	1.10	0.74 (0.52, 1.05)	0.09	0.78 (0.65, 0.94)	0.01
≧4	81	3928	2.06	59	4310	1.37	0.67 (0.48, 0.94)	0.02	0.70 (0.57, 0.85)	0.02
Comorbidity ^†^										
Yes	219	13,065	1.68	168	13700	1.23	0.74 (0.61, 0.91)	0.004	0.78 (0.70, 0.87)	0.001
No	26	1660	1.57	20	1519	1.32	0.79 (0.44, 1.42)	0.43	0.81 (0.56, 1.16)	0.24

PY, person-years; IR, incidence rate, per 100 person-years; HR, hazard ratio; ^†^ Patients with any one of comorbidity were classified as the comorbidity group; CI, confidence interval; Models adjusted by gender, age, DCSI score, and all comorbidities and medications are listed in Table 1.

**Table 3:** Incidence rate and hazard ratio of allergic rhinitis in diabetes patients by cumulative dose of DPP-4 inhibitor use compared to non-users.

Variable	N	Event	PY	IR	Crude		Adjusted	
					HR (95% CI)	*p*-Value	HR (95% CI)	*p*-Value
Non-users	6204	245	14,725	1.66	1.0		1.0	
DPP-4 inhibitor use, cDDDs								
≦279	2046	112	4097	2.73	1.64 (1.31, 2.05)	<0.0001	1.87 (1.65, 2.11)	<0.0001
280–647	2073	50	4373	1.14	0.68 (0.50, 0.93)	0.01	0.75 (0.64, 0.88)	0.01
≧648	2085	26	6749	0.39	0.24 (0.16, 0.35)	<0.0001	0.25 (0.20, 0.31)	<0.0001

PY, person-years; IR, incidence rate, per 100 person-years; Models adjusted by gender, age, DCSI score, and all comorbidities and medications listed in Table 1. HR, hazard ratio; CI, confidence interval; cDDD, cumulative defined daily dose.

**Table 4:** Cox proportional hazard regression analysis for the interaction of other oral antidiabetic agents and DPP-4 inhibitors on allergic rhinitis risk.

Variables		*N*	Event	Adjusted HR(95% CI)	*p*-Value ^&^
DPP-4 inhibitors	Thiazolidinedione				<0.001
No	No	5890	240	1 (Reference)	
Yes	No	5233	158	0.76 (0.69, 0.85)	
No	Yes	314	5	0.32 (0.20, 0.52)	
Yes	Yes	971	30	0.69 (0.56, 0.85)	
DPP-4 inhibitors	Sulfonylureas				0.29
No	No	5566	228	1 (Reference)	
Yes	No	5230	165	0.79 (0.71, 0.88)	
No	Yes	638	17	0.75 (0.57, 0.99)	
Yes	Yes	974	23	0.76 (0.59, 0.99)	
DPP-4 inhibitors	Biguanides				<0.001
No	No	5302	227	1 (Reference)	
Yes	No	5058	155	0.73 (0.65, 0.82)	
No	Yes	902	18	0.53 (0.40, 0.69)	
Yes	Yes	1146	33	0.90 (0.72, 1.14)	
DPP-4 inhibitors	Other antidiabetic drugs				0.002
No	No	5713	234	1 (Reference)	
Yes	No	4779	150	0.77 (0.70, 0.86)	
No	Yes	491	11	0.47 (0.34, 0.65)	
Yes	Yes	1425	38	0.63 (0.52, 0.76)	

Models adjusted by gender, age, DCSI score, and all comorbidities and medications listed in Table 1. ^&^ *p*-value for interaction.
